# Research advances and challenges in tissue-derived extracellular vesicles

**DOI:** 10.3389/fmolb.2022.1036746

**Published:** 2022-12-15

**Authors:** Zhengke Zhi, Qiaochu Sun, Weibing Tang

**Affiliations:** Department of Pediatric Surgery, Children’s Hospital of Nanjing Medical University, Nanjing, China

**Keywords:** extracellular vesicles, tissue derived extracellular vesicles, microenvironment, biomarker, tumor

## Abstract

Extracellular vesicles (EV) are vesicular vesicles with phospholipid bilayer, which are present in biological fluids and extracellular microenvironment. Extracellular vesicles serve as pivotal mediators in intercellular communication by delivering lipids, proteins, and RNAs to the recipient cells. Different from extracellular vesicles derived from biofluids and that originate from cell culture, the tissue derived extracellular vesicles (Ti-EVs) send us more enriched and accurate information of tissue microenvironment. Notably, tissue derived extracellular vesicles directly participate in the crosstalk between numerous cell types within microenvironment. Current research mainly focused on the extracellular vesicles present in biological fluids and cell culture supernatant, yet the studies on tissue derived extracellular vesicles are increasing due to the tissue derived extracellular vesicles are promising agents to reflect the occurrence and development of human diseases more accurately. In this review, we aimed to clarify the characteristics of tissue derived extracellular vesicles, specify the isolation methods and the roles of tissue derived extracellular vesicles in various diseases, including tumors. Moreover, we summarized the advances and challenges of tissue derived extracellular vesicles research.

## 1 Introduction

Extracellular vesicles (EVs) are nano lipid bilayer-delimited particles released by a variety of cells (Yanez-Mo et al., 2015). Microvesicles (MVs), exosomes, and apoptotic bodies are the three major subtypes of EVs, which are characterized mainly based on their size, biological properties, and production process (Crescitelli et al., 2013; Shao et al., 2018). In brief, MVs are released by cellular membrane budding with 100–1,000 nm in size, while exosomes (30–150 nm) are formed by the fusion of multivesicular bodies (MVBs) and cell membrane, and apoptotic bodies are delivered during the process of cell apoptosis with a diameter of 500–2000 nm (Raposo and Stoorvogel, 2013; DeLeo and Ikezu, 2018; Gurunathan et al., 2019). However, the three EV subtypes are difficult to distinguish thoroughly because of the overlap in size (Marar et al., 2021). Therefore, they are frequently classified as small, medium, and large EVs according to their size. All lipid-bilayer particles released by cells will be referred to as “EVs” in this review in accordance with the International Society for Extracellular Vesicles (ISEV) nomenclature, with size designations where necessary (Thery et al., 2018).

Before being released, EVs can package multiple lipids, nucleic acids and proteins. Owing to the stable lipid membrane of EVs, these cargoes are prevented from degradation (Andaloussi et al., 2013; Gupta et al., 2021). When EVs are delivered into the extracellular environment, they can directly bind to the surface of recipient cells and subsequently initiate intracellular signaling pathways or deliver their cargoes to the recipient cells through endocytosis (Valadi et al., 2007; Raposo and Stoorvogel, 2013; Mulcahy et al., 2014; Xiao et al., 2014). As a result, by acting as messengers in cell-to-cell communication, EVs play a critical role in various physiological and pathological processes, as well as the morbidity and development of numerous diseases (Mateescu et al., 2017; Hill, 2019; Raposo and Stahl, 2019; Russell et al., 2019; Urabe et al., 2020). For example, the EVs produced by tumor cells encourage the invasion and metastasis of ovarian cancer by delivering miR-106a-5p to recipient cells to target KLF6 (Zheng et al., 2022). Exosomal miR-146a-5p and miR-155-5p communicate with cancer-associated fibroblasts to promote CXCL12/CXCR7-induced metastasis of colorectal cancer (Wang et al., 2022). In wound healing, several studies proved that EVs derived from mesenchymal stromal cells (MSCs) are conducive to assist wound closure (Zhang et al., 2015; Ha et al., 2016). Furthermore, Sun et al. (Sun et al., 2022) found EVs are key mediators during the early hypoxia-induced damage signaling transduction from endothelial cells to cardiomyocytes in acute myocardial infarction (AMI).

EVs widely exist in almost all types of body fluids, including blood, urine, cerebrospinal fluid (CSF), saliva and tears. Thus they are appropriate biomarkers for the early diagnosis and prognosis in clinical application due to these body fluids are relatively easily to collect (Keller et al., 2011; Lasser et al., 2011; Muraoka et al., 2020a; Fringuello et al., 2021; Yu et al., 2021; Liu et al., 2022). As reported, the circulating miR-135a-3p in serum EVs has been characterized as a possible biological marker of non-alcoholic fatty liver disease (Jiang et al., 2021a). In Parkinson’s disease (PD), serum EVs-derived miRNAs (miR-374a-5p, miR-374b-5p, miR-199a-3p, miR-28-5p, miR-22-5p and miR-151a-5p) are promising biomarkers for PD progression and early diagnosis (He et al., 2021). Recently, a database (exoRBase 2.0) has been established to display the atlas of mRNAs and non-coding RNAs in EVs from human biological fluids, which will be beneficial for discovering novel circulating biomarkers to augment the diagnosis and therapy of human diseases (Lai et al., 2022).

Nowadays, researches on EVs have expanded from cells and body fluids to the characterization of EVs from the extracellular spaces within tissues (Perez-Gonzalez et al., 2012; Vella et al., 2017; Li et al., 2021). Increasing evidence have shown the EVs isolated from tissues possess more abundant biological information and are more accurately to reflect the alteration of tissue microenvironment compared to those derived from cells and biological fluids (Asai et al., 2015; Jang et al., 2019). These studies mainly focused on the tissue derived EVs (Ti-EVs) extracted from whole tissues (Perez-Gonzalez et al., 2012; Silverman et al., 2019; Lasser et al., 2021), or short-term culture of tissue explants, such as *ex vivo* tumors (Mincheva-Nilsson et al., 2016; Lunavat et al., 2017). In this review, we aimed to make a summary of the studies on Ti-EVs in recent years to provide a better understanding of the characteristics and potential values of EVs derived from tissues.

## 2 Perspective of EVs derived from tissues

In the past decade, a variety of studies about EVs isolated from *in vitro* cell culture systems and body fluids have been reported (Nielsen et al., 2017; Breglio et al., 2020; Penas-Martinez et al., 2021). However, the *in vitro* cell culture environment is a relatively unitary system and cannot fully simulate the complex intrinsic microenvironment. Besides, the process of long-term cell cultivation can change the cell characteristics, and subsequently influence the EVs released from cell lines (Allen et al., 2016; Crescitelli et al., 2020). More important, the cell lines may not be accurately authentic to the tissues of origin. By employing gene profiling and transcriptome analysis in human glioma cell lines, Allen et al. (Allen et al., 2016) found the DNA profile of a commonly used glioma cell line U87MG was distinct from that of the original cells. On the contrary, it might be a cell line of uncertain origin. Through the analysis of more than 40 ovarian cancer cell lines, it was shown that high-grade serous ovarian cancer sample tissues and commonly utilized ovarian cancer cell lines had significantly different molecular profiles, indicating that cell lines are not completely consistent with tissues (Domcke et al., 2013). Besides, although the EVs isolated from biological fluids contain a great deal of information, they are a very mixed collection from multifarious cells and organs (De Wever and Hendrix, 2019; Huang and Xu, 2021). Therefore, the abundant information is confused and it is also difficult to determine the origin of these fluids derived-EVs.

**FIGURE 1:**
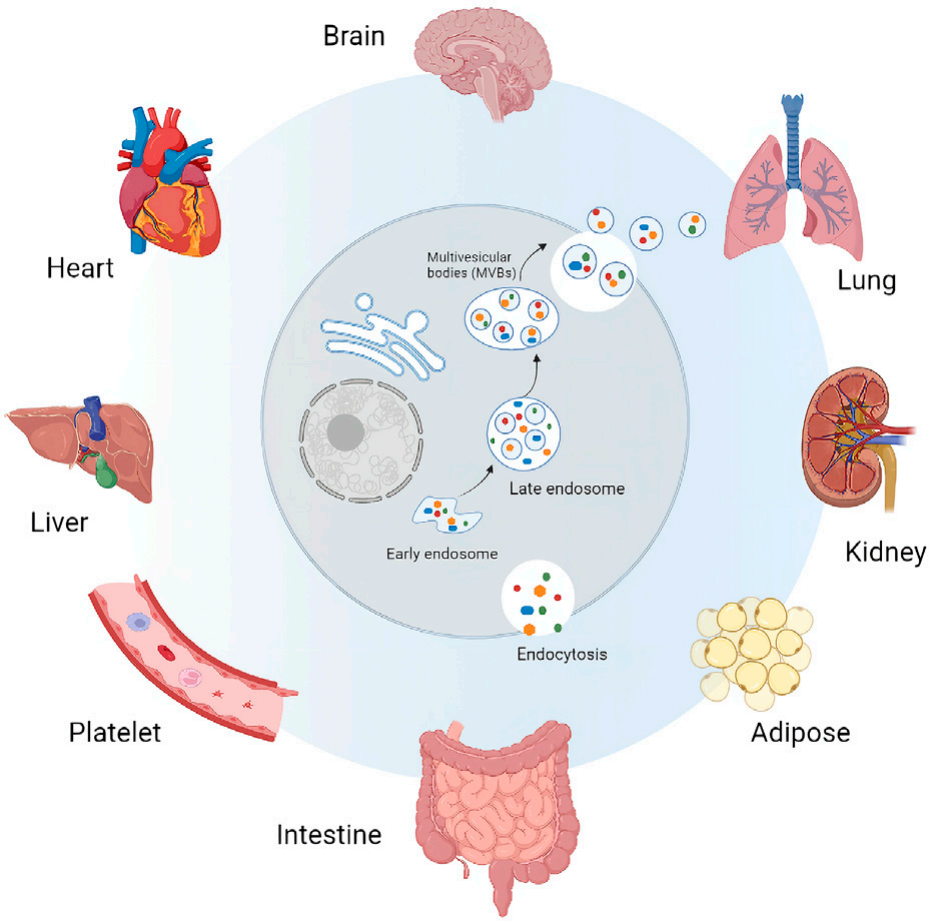
Sources of tissue derived extracellular vesicles (Ti-EVs). Ti-EVs have been isolated from brain, heart, liver, lung, kidney and intestine tissues, as well as adipose tissue and platelet.

Compared with the cell culture and body fluids derived EVs, the Ti-EVs can carry more original information, and reflect the intercellular communication more truly (Table 1) (Camino et al., 2020). In addition, the source of Ti-EVs is more clear, so they are easier to track. For instance, Ti-EVs can be directly visualized in the interstitial space of a melanoma metastatic tissues under the electron microscopy, suggesting these Ti-EVs might originate from the metastatic foci (Jang et al., 2019). By using the immuno-TEM (Transmission Electron Microscopy), several nanosized (30–100 nm in diameter) EVs which were characterized by the round shape, are observed clearly in the cytoplasm of human benign prostatic hyperplasia (BPH) cells and prostate cancer cells in the tumor microenvironment (Park et al., 2016). Owing to the above reasons, to isolate EVs from different types of tissues (such as brain, heart, lung, and kidney), and explore their roles in multiple cellular process has gained more and more attention (Figure 1) (Li et al., 2021; Chang et al., 2022).

**TABLE 1:** Characteristics of Ti-EVs.


Sources	Whole tissues (diverse tumor tissues, brain, heart, e.g.) Crescitelli et al. (2020); Gallart-Palau et al. (2016); Muraoka et al. (2020c); Ge et al. (2019)
	Tissue explants (*ex vivo* tumors, e.g.) Mincheva-Nilsson et al. (2016); Lunavat et al. (2017)
	Other tissues (such as adipose and plaque, e.g.) Packer (2018); Zhang et al. (2017)
Advantages	Carry more abundant biological information
	Reflect the alteration of tissue microenvironment more accurately Asai et al. (2015); Jang et al. (2019)
	Contain more original information than cell lines and body fluids
	More traceable Jang et al. (2019); Park et al. (2016)
Application	Reliable biomarkers for tumor diagnosis and prognosis Himbert et al. (2020); Huang et al. (2017); Liang et al. (2021)
	Promising regulator of neurodegenerative disorder Guo et al. (2016); Arai et al. (2006)
	Potential treatment for cardiac diseases Hausenloy and Yellon (2013)

## 3 Methods for extracting Ti-EVs

**FIGURE 2:**
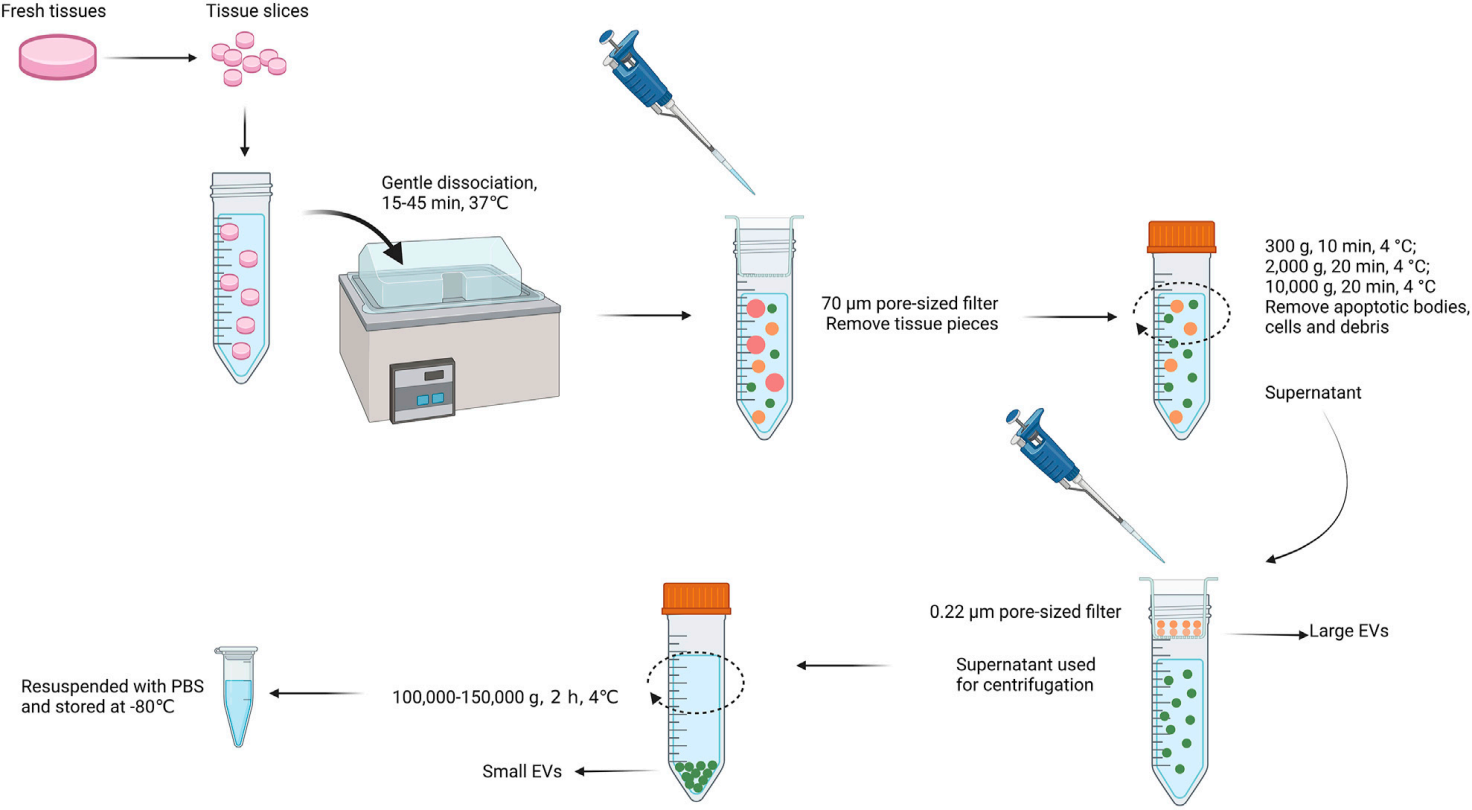
The promising universal method applied for extracting Ti-EVs from a variety of tissues. At first, fresh tissues are sliced into slices and subsequently dissociated gently for 15–45 min at 37°C. Afterwards, a 70 μm pore-sized filter is used to remove tissue pieces. Centrifugation at a speed of 300–10,000 g at 4°C is conducted to remove apoptotic bodies, remaining cells and debris and the supernatant pass through a 0.22 μm filter to collect large EVs. Next, the filtrate is ultracentrifugated at a speed of 100,000–150,000 g at 4°C (>1 h) to collect small EVs. Finally, the isolated Ti-EVs are resuspended with PBS and can be stored at −80°C for a long time.

Currently, EVs studies are limited by the shortage of EVs isolation and purification methods (Ramirez et al., 2018; Royo et al., 2020). Up to date, several methods of EVs isolation and purification are commonly used, including differential centrifugation (DC) (Tauro et al., 2013; Pospichalova et al., 2015), ultracentrifugation (UC) (Gardiner et al., 2016), density gradients centrifugation (DGC) (Jeppesen et al., 2019), microfluidic devices (Liga et al., 2015; Konoshenko et al., 2018), size exclusion chromatography (SEC), synthetic polymer–based precipitation (Van Deun et al., 2014), and membrane filtration(UF) (Grant et al., 2011). Even though, they all have limitations, because the size and physicochemical properties of EVs always overlap with lipoproteins, chyle particles and so on. There is still a lack of methods focusing on the separation of tissue derived EVs (Cianciaruso et al., 2019; Huang et al., 2020). Furthermore, how to isolate EVs from tissues but not damage the cell membranes so that to maintain the native shape and function of Ti-EVs during the process of extraction is one of the grand challenges (Thery et al., 2018). Several published methods for isolating EVs from tissues usually involve homogenization and filtration, which can mix the extracellular environment with intracellular vesicles as well as other nanosized particles (Perez-Gonzalez et al., 2012; Gallart-Palau et al., 2016). In order to dissociate the tissues without disrupting cell, and improve the purity of Ti-EVs isolation, the method of combining the high-speed centrifugation, discontinuous sucrose gradient ultracentrifugation, filtration, and following with ultracentrifugation is recommended (Muraoka et al., 2020b; Crescitelli et al., 2021; Ruan et al., 2021). Another possible approach is utilizing collagenase (e.g. type I and III) to dissociate cells from human and animal tissues before centrifugation. This method is applicable for the tissues that are sensitive to damage, such as brain tissues, primary and metastatic tumor tissues (Vella et al., 2017; Crescitelli et al., 2020). Figure 2 illustrates a promising universal method applied for extracting Ti-EVs from diverse tissues (Crescitelli et al., 2021). Besides, extraction of EVs from fresh tissues rather than frozen tissues are more conducive to the purification of Ti-EVs (Crescitelli et al., 2020). This may be because the freezing process will cause damage to cells, resulting in cell rupture and overflow of contents. Although some strategies for separating Ti-EVs have been developed, researchers need to make appropriate adjustments to the separation method based on the type of tissue samples because of the complexity and specificity of different tissues.

## 4 Ti-EVs in human diseases

### 4.1 Ti-EVs in tumors

In human cancers, significant progress has been made in the study of cell culture and bio-fluid derived EVs (Becker et al., 2016; Weng et al., 2021; Namee and O'Driscoll, 2018; Vaidyanathan et al., 2018). Despite that, increasing evidence has revealed the critical roles of Ti-EVs in multiple tumors (Table 2) (Cianciaruso et al., 2019; Himbert et al., 2020). For example, after collecting 426 human samples, including tissue explants, plasma, and other bodily fluids, the proteomic profile of EVs was conducted to develop improved biopsy method for the detection of cancers. Interestingly, the comparison of Ti-EVs identified proteins that distinguish tumors from normal tissues with a sensitivity of 90% and a specificity of 94%, suggesting that Ti-EVs are reliable biomarkers for the examination of cancer and determination of cancer type (Hoshino et al., 2020).

**TABLE 2:** The role of Ti-EVs in diverse tumor tissues.

Tumors	Value of Ti-EVs	Key factors	Potential application
Melanoma Jang et al. (2019)	Abundant mitochondrial membrane protein	MT-CO2, COX6c	Predictor for the occurrence of melanoma
Clear cell renal carcinoma Cianciaruso et al. (2019)	Tumor-specific EVs	CD147, CA9, CD70	Potential biomarker
Colorectal cancer Himbert et al, (2020)	Associated with inflammation and lipid metabolism	STING, TLRs, COX1, TBXAS1	Modulating tumor immune microenvironment
Gastric cancer Zhang et al. (2018)	Inducing autophagy and NF-κB pathway	HMGB1, TLR4	Reshaping tumor microenvironment
Pancreatic ductal adenocarcinoma Liang et al. (2021)	Maintaining the hepatic fibrosis microenvironment, and the liver metastasis	CD44v6, C1QBP	Predictor for the prognosis and liver metastasis
Urothelial bladder cancer Eldh et al. (2021)	Associated with the cancer metabolic pathways	PGK1, ALDOA, GSTP1	Potential biomarker

Malignant melanoma is the most aggressive and life-threatening skin cancer which originates from melanocytes (Cheng et al., 2021). In melanoma, EVs isolated from tumor tissues were found different from the classical cell line-derived EVs. These Ti-EVs have more abundant mitochondrial membrane proteins, and can be applied as potential predictors for the occurrence of melanoma (Jang et al., 2019). Zhang et al. (2018) demonstrated that tumor tissue-derived EVs from gastric cancer could induce higher expression of autophagy related genes ATG7 and BECN1, as well as the activation of NF-κB signaling pathway in neutrophils than EVs derived from non-cancerous tissues. Furthermore, neutrophils treated with EVs isolated from gastric cancer tissues promoted the migration of gastric cancer cells more effectively. Pancreatic ductal adenocarcinoma (PDAC) is a malignant type of pancreatic cancer (Siegel et al., 2021). Because of the insufficient understanding about the occurrence of liver metastasis in PDAC, the options for the therapeutic treatment of PDAC liver metastasis are very limited (Garrido-Laguna and Hidalgo, 2015; Huang et al., 2017). Nevertheless, the EVs derived from PDAC have been confirmed to mediate the crosstalk between the primary PDAC cells and hepatic satellite cells (HSCs). PDAC-EVs carrying CD44v6/C1QBP are essential for maintaining the hepatic fibrosis microenvironment and the liver metastasis of PDAC, indicating that EVs-CD44v6/C1QBP are potential biomarker for the prediction of the prognosis and liver metastasis of PDAC patients (Liang et al., 2021). Muscle-invasive urothelial bladder cancer (UBC) is a malignancy characterized by poor prognosis and high morbidity (Chamie et al., 2013). In order to explore new appropriate biomarkers for the occurrence and development of UBC, the Ti-EVs were extracted from UBC tumor tissues and matched distant tissues for further proteomics analysis. The results showed that 69 most abundant proteins profiled in tissue-derived EVs, regardless of the sites close to or away from the original tumor, were closely associated with the cancer metabolic pathways and poor prognosis (Eldh et al., 2021). These findings confirm the release of malignant EVs in UBC even though the pathologically undetectable tumor type, thus emphasizing the necessity of early radical cystectomy for UBC patients. At present, suitable diagnostic biomarkers for clear cell renal carcinoma (ccRCC) are still inadequate (Wang et al., 2018). To identify appropriate tumor-specific exosomal markers in ccRCC, the expression of CD147 (a pan-cancer-specific protein), CD70 and CA9 (the well characterized proteins in ccRCC) (Jilaveanu et al., 2012; Li et al., 2017; Baniak et al., 2020; Peng et al., 2020) in EVs were examined. Highly expressed CA9, CD70, and CD147 were found in Ti-EVs from tumor tissues compared to that in normal tissues. Therefore, CA9, CD70, and CD147 might function as potential biomarkers for the identification of tumor-specific EVs in ccRCC (Himbert et al., 2020).

Tumor metastasis is a very challenging problem. Recent evidence suggests that there is a complex communication mechanism between primary tumors and metastases, whereas primary tumors can influence the microenvironment of distant organs to induce metastasis (Kaplan et al., 2005; Hara et al., 2017; Ji et al., 2020). The microenvironment modified by primary tumors is referred as the metastatic niche which is characterized by vascular permeability, extracellular matrix remodeling, bone marrow-derived cells recruitment, angiogenesis, and immunosuppression (Dong et al., 2021; Garcia-Silva et al., 2021). EVs secreted by primary tumors are pivotal mediators during the formation of metastatic niche by delivering agents with tumor characteristics to the recipient cells in distant organs (Li et al., 2019a; Lee et al., 2019; Mashouri et al., 2019). For example, the EVs derived from ovarian primary tumor directly promote circulating tumor cells homing, colonization, and outgrowth within the metastatic niche but inhibit the antitumor immune response of host mircoenvironmentt (Feng et al., 2019). In salivary adenoid cystic carcinoma, primary tumor derived EVs activate lung fibroblast and induce the formation of lung metastatic niche which subsequently accelerate the lung metastasis (Kong et al., 2019). Moreover, it was found that EVs derived from highly metastatic murine breast cancer were primarily absorbed by the lungs of mice, and the T-cell proliferation and NK cell cytotoxicity were inhibited obviously. This may explain why the microenvironment in distant organs are always immunosuppressive (Wen et al., 2016; Patel et al., 2018). In summary, the Ti-EVs derived from tumors are tightly involved in the progress and metastasis of cancers, and they are potential targets for developing new diagnostic and therapeutic methods for tumors.

### 4.2 Ti-EVs in other diseases

#### 4.2.1 Ti-EVs in neurodegenerative disorders

Neurodegenerative disorders are a group of central nervous system (CNS) diseases related to brain function degeneration, including Alzheimer’s Disease (AD), Parkinson’s Disease (PD), Amyotrophic Lateral Sclerosis (ALS) and Huntington’s Disease (Gao et al., 2021). Alzheimer’s disease (AD) is the most common neurodegenerative disorder, which is characterized by the amyloid plaques and the intracellular accumulation of neurofibrillary tangles (NFTs) in brain tissue (Goedert et al., 1988; Duyckaerts et al., 2009). The role of EVs in AD has been widely studied, however, the EVs from brain tissues have not been well explored (Hill, 2019; Nogueras-Ortiz et al., 2020). Tau and Aβ oligomers are the most AD related pathogenic proteins which have also been detected in brain tissue derived EVs (BDEVs), indicating that BDEVs might participate in the AD pathogenesis (Guo et al., 2016; DeLeo and Ikezu, 2018). With the deepening of research, a quantitative proteomic analysis of the BDEVs isolated from AD patients and control ones was conducted. The proteomic analysis elucidated that ANXA5, GPM6A, VGF, and ACTZ could distinguish AD EVs from controls with high accuracy, thus providing novel biomarkers for AD (Muraoka et al., 2020c). Moreover, by isolating BDEVs from the frontal cortex of AD patients and control subjects, Cheng et al. (2020) found that BDEVs derived from AD patients contain more disease-associated miRNAs. Although there are few studies about the interplay of RNAs in BDEVs, the above findings will be beneficial for clarifying the early pathological changes of AD. Amyotrophic lateral sclerosis (ALS) is a neurodegenerative disease caused by the deposition of ubiquitinated and aggregated proteins in lewy body-like hyaline, or skein-like inclusions in the upper and lower motor neurons cytoplasm (Taylor et al., 2002; Arai et al., 2006). In order to explore the potential roles of Ti-EVs in ALS, the motor cortex derived EVs (MCEVs) were isolated from the brain tissues of ALS patients and neurological controls to perform proteomic analysis. As the result of proteomic analysis shown, 16 proteins (including several stress granule dynamics related proteins, such as STAU1 and DHX30) were found to be differentially expressed in ALS MCEVs compared to that of controls, suggesting that these MCEVs are promising targets for treating ALS. Moreover, the finding of these proteins in MCEVs reveals that ALS related stress particles might be tightly involved in the package of MCEVs thereby highlighting the indispensability of EVs in the pathogenesis of ALS (Vassileff et al., 2020). Parkinson’s Disease (PD) is an important member of neurodegeneration, and some studies have elucidated the role of EVs in PD (Leggio et al., 2021; Huang et al., 2022). However, the research on brain derived EVs in PD is still insufficient, and more in-depth research is necessary. In addition, further studies are needed to detect these brain tissue-EVs relevant proteins and RNAs in EVs from CSF and blood, so as to evaluate their potential value as diagnostic markers.

#### 4.2.2 Ti-EVs in cardiac disease

In acute myocardial infarction (AMI), the reperfusion can induce irreversible myocardial injury, which is also known as myocardial ischemia-reperfusion (IR) injury (Hausenloy and Yellon, 2013). To date, the underlying mechanism of IR injury remains unclear. As reported, the cardiac IR induced EVs could accelerate IR injury. A novel IR-EVs-miR-155-5p-M1 polarization pathway was found in heart tissue during the progression of IR, shedding lights on the therapeutic potential of cardiac Ti-EVs for the treatment of IR injury (Ge et al., 2021). By establishing mice IR model, Zhou et al. extracted Ti-EVs from the IR heart tissues, and illustrated a specific profile of circRNAs in Ti-EVs, providing evidences about the role of Ti-EVs in the development of cardiac IR (Ge et al., 2019). When the acute myocardial infarction occurs, a number of inflammatory cells will be rapidly recruited to the ischemic areas, leading to the massive release of cytokines, soluble chemokines, and growth factors (Silvestre et al., 2013). By comparing the Ti-EVs isolated from the heart tissues of coronary artery ligation and sham mice. It was found that AMI increased the release of cardiac EVs which were immediately absorbed by infiltrating monocytes and then to modulate the local inflammatory responses (Loyer et al., 2018). These findings indicated that the local Ti-EVs formation in the infarcted heart are closely related to the inflammation after myocardial infarction.

## 5 Other origins of Ti-EVs

### 5.1 Adipose tissue

Adipose tissue (AT) has been regarded as an endocrine organ, which can secrete adipokines, such as proinflammatory cytokines, anti-inflammatory cytokines and metabolism regulatory cytokines (Cui et al., 2017; Packer, 2018; Wei et al., 2020). Adipose tissue can release EVs and these EVs are considered to be the main source of circulating EVs (Wei et al., 2020). In adipose tissue-secreted EVs (AT-EVs), abundant miRNAs have been detected, suggesting that AT-EVs may convey intercellular information and signals (Thomou et al., 2017; Lee et al., 2021). As reported before, the obese mice could release enriched AT-EVs (derived from perivascular adipose tissues, PVAT) which packed miR-221-3p. The receipt of these EVs in lean mice aroused the response of inflammation in PVAT and vascular phenotypic switching in abdominal aorta, highlighting the importance of AT-EVs in cellular crosstalk to participate obesity-associated inflammation (Li et al., 2019b). Adipose tissue-derived stem cells (ADSCs) can internalize the EVs derived from adipose tissues and induced adipogenesis. Mechanically, AT-EVs reduced the expression of adipogenesis related protein WISP2 in ADSCs by transferring miR-450a-5p to them (Zhang et al., 2017). Brown adipose tissue (BAT) is a major kind of adipose tissue. Interestingly, the sEVs secreted by BAT are involved in exercise cardioprotection *via* delivering the cardioprotective miRNAs into the heart. This result showed the BAT-sEVs are critical mediator in the interaction between BAT and cardiomyocyte (Zhao et al., 2022). Besides, AT-EVs obtained from gestational diabetes mellitus (GDM) and normal glucose tolerant (NGT) have been confirmed to have differently expressed proteins, which may contribute to fetal overgrowth in GDM (Jayabalan et al., 2019).

### 5.2 Plaque

The stability and severity of the plaque is closely related to the crosstalk between itself and the microenvironment. Electron microscopic analysis of ultrathin sections has verified the presence of EVs in human atherosclerotic plaque, indicating that lesional smooth muscle cells (SMCs) and endothelial cells within the plaque are able to release EVs into the extracellular space. This finding provides a novel aspect of intercellular communication in plaque environment (Perrotta and Aquila, 2016). In another study about metabolic syndrome (MetS), EVs isolated from the atherosclerotic plaques of mouse and human both contained abundant Rap1, which is crucial for the inflammatory response of vascular (Metrich et al., 2010). Researchers investigated the excessive production of Ti-EVs enriched in Rap1 could promote the development of atherosclerosis in MetS by inducing the remodel and inflammation of vascular (Perdomo et al., 2020). Nicotine is the cigarette smoke’s main component which could directly stimulate plaque cell migration and proliferation as well as the communication of cytokines between macrophages and VSMCs *via* EVs, thus accelerating atherogenesis (Liu et al., 2017; Ren et al., 2018). Furthermore, nicotine could facilitate atherosclerotic lesion progression and cause plaque derived EVs to remain *in vivo*, which might mediate the migration and proliferation of VSMC (Zhu et al., 2019).

### 5.3 Cochlea

In the inner ear, cochlea is a vital organ that responsible for the auditory signal transduction. Cochlea develops during the period of embryonic day 9 to postnatal day 21 (Wright et al., 2003; Burns et al., 2012). Both the detection of sound waves and the transmission of sound information to the brain are relied on cochlear hair cells (HCs) (LeMasurier and Gillespie, 2005). In spite of the widely research about EVs in cancer and some other diseases, there are few studies to reveal the role of EVs in cochlea. Lately, (Jiang et al. (2022) has isolated cochlear tissue-derived EVs from mice of different ages to analyze and characterize the protein and miRNA contents of EVs. More than five hundred miRNAs and five thousand proteins have been detected in the EVs derived from cochlear. Among them, about two hundred miRNAs and three thousand proteins are expressed differentially at different periods, which are probably involved in the maturation of HCs. These findings authenticated that EVs are present in the cochlea tissues and important for the development of auditory system. Meanwhile, these EVs-miRNAs and proteins are promising novel targets for studying the mechanism of cochlear development.

## 6 Conclusion and future directions

Compared with EVs isolated from cell culture supernatant and body fluids, Ti-EVs may play a more crucial role in different sorts of diseases. Due to Ti-EVs are located in the tissue microenvironment, they have abundant cell crosstalk information and can authentically reflect the intercommunication between cells within tissue microenvironment. Moreover, the contents of Ti-EVs are relative pure because of its single tissue source. Based on these advantages, Ti-EVs have attracted increasing attention and have been studied a lot in basic research and clinical applications. Although some progress has been achieved, there are still some challenges and limitations needed to overcome. Firstly, Ti-EVs isolated by existing methods are often contaminated by EVs released from other broken cells. Therefore, it is urgent to develop optimal techniques to isolate and purify Ti-EVs. Furthermore, the tissue microenvironment contains diverse cell types, to elucidate the accurate original cell type of Ti-EVs will provide deep understanding for the intercellular communication within microenvironment. To further investigate the chemical, physical, and biological characteristics of Ti-EVs may help us distinguish Ti-EVs from “impurities”. Secondly, some researches have demonstrated the potential of Ti-EVs to be diagnostic biomarkers, however, the isolation methods are too complex to apply in clinical practices and the exploration of Ti-EVs in the pathophysiological process of various diseases are still in the initial stage. How to balance the purity and convenience of Ti-EVs extraction is a major challenge in the future. Thirdly, considering the spatiotemporal specificity of tissue development, to illustrate the spatiotemporal profiles of Ti-EVs in all kinds of tissues will help us understand the development process deeply.

Although the widely clinical application of EVs is still difficult because of the problems in EVs preparation, transmission, targeting and retention, some achievements have been made. A variety of studies have shown diverse biomaterials for delivering EVs efficiently, including implantable scaffolds (Jiang et al., 2021b), injectable hydrogels (Wong et al., 2020), and chemical crosslinking (Heirani-Tabasi et al., 2021). Modifying EVs to meet different needs is another strategy. These engineered EVs can carry cell targeted ligands and a high level of specific agents, which are expected to improve their therapeutic effects, especially in multiple tumors (Zhan et al., 2020; Zhao et al., 2020; Wong et al., 2021). In addition, to insert imaging molecules in engineered EVs is an appropriate method for determining the metabolism and distribution of EVs *in vivo* (Luan et al., 2017; Liang et al., 2021).

Without a doubt, a deeper comprehension of Ti-EVs biology and the development of standardized techniques for Ti-EVs quantification, extraction and storage, molecular characterization, and potency assays will greatly advance the prospects of Ti-EVs-based diagnostic and therapeutic applications in the future. Challenges do exist at present, but with the improvement of technology and development of research, these difficulties will eventually be overcome. The clinical application of Ti-EVs will gradually come true.
